# Extracellular vesicles from a muscle cell line (C2C12) enhance cell survival and neurite outgrowth of a motor neuron cell line (NSC-34)

**DOI:** 10.3402/jev.v3.22865

**Published:** 2014-02-19

**Authors:** Roger D. Madison, Christopher McGee, Renee Rawson, Grant A. Robinson

**Affiliations:** 1 Department of Surgery, Duke University Medical Center, Durham, NC, USA; 2 Research Service of the Veterans Affairs Medical Center, Durham, NC, USA

**Keywords:** extracellular vesicles, motor neuron, muscle, neurite outgrowth

## Abstract

**Introduction:**

There is renewed interest in extracellular vesicles over the past decade or 2 after initially being thought of as simple cellular garbage cans to rid cells of unwanted components. Although there has been intense research into the role of extracellular vesicles in the fields of tumour and stem cell biology, the possible role of extracellular vesicles in nerve regeneration is just in its infancy.

**Background:**

When a peripheral nerve is damaged, the communication between spinal cord motor neurons and their target muscles is disrupted and the result can be the loss of coordinated muscle movement. Despite state-of-the-art surgical procedures only approximately 10% of adults will recover full function after peripheral nerve repair. To improve upon such results will require a better understanding of the basic mechanisms that influence axon outgrowth and the interplay between the parent motor neuron and the distal end organ of muscle. It has previously been shown that extracellular vesicles are immunologically tolerated, display targeting ligands on their surface, and can be delivered in vivo to selected cell populations. All of these characteristics suggest that extracellular vesicles could play a significant role in nerve regeneration.

**Methods:**

We have carried out studies using 2 very well characterized cell lines, the C2C12 muscle cell line and the motor neuron cell line NSC-34 to ask the question: Do extracellular vesicles from muscle influence cell survival and/or neurite outgrowth of motor neurons?

**Conclusion:**

Our results show striking effects of extracellular vesicles derived from the muscle cell line on the motor neuron cell line in terms of neurite outgrowth and survival.

Classical neuroscience experiments carried out almost 40 years ago demonstrated that if a foreign nerve was sutured to a muscle, axons from the foreign nerve would grow over the surface of the normally innervated muscle but they would not enter the muscle and form neuromuscular junctions. However, shortly after denervation of the original nerve that innervated the muscle, motor axons from the foreign nerve would now enter the muscle and form functional neuromuscular contacts (1, 2). This suggests that the acutely denervated muscle was secreting signals, operating over some distance, that successfully directed the foreign nerve fibres to neuromuscular junctions. More recently it has been shown that several neurotrophic factors are indeed increased in muscle due to denervation (e.g. HGF, FGFs, BDNF) (3–9), and that the increased endocytotic/exocytotic activity that has been observed occurs predominantly at the endplate region of the muscle (10). These observations have led to the suggestion that the high exocytotic activity of denervated muscle is due to the increased secretion of various factors by the muscle in an attempt to stimulate reinnervation by regenerating axons of motor neurons (11–15). There is also a vast amount of data that soluble extracts of conditioned media from muscle can act as a trophic factor for motor neurons (16). Given such well-known influences of muscle on motor neuron development and regeneration (e.g. see Ref. (17) for a classic review), we have begun to investigate the possibility that, in addition to signals from the soluble fraction, extracellular vesicles derived from muscle cells may stimulate survival and neurite outgrowth from motor neurons.

The term extracellular vesicles is quite inclusive, generally referring to exosomes (<100 nm) and microvesicles, ectosomes, or shed vesicles (>100 nm) (18). We have adopted this general nomenclature for the present work while being well aware that future work in the field may lead to more specific categories based on selected characteristics of such vesicles. Nanometer-sized secreted vesicles were originally described more than 30 years ago and were thought to act as cellular “garbage cans” containing unwanted molecular components; they remained little studied for at least 10 years. Over the past few years there has been an explosive interest in these small vesicles because of the discovery that they become a protected environment for various forms of RNA as well as proteins and thus function as a novel mechanism for long-distance intercellular signalling. This unique function was confirmed when it was shown that secreted extracellular vesicles containing mRNA from one cell type can be translated into protein in recipient target cells (19). A current PubMed search now reveals several thousands of articles about extracellular vesicles, with most of them occurring in the last 4–5 years. Extracellular vesicles are secreted by just about every cell type that has been examined, including tumour cells, reticulocytes, epithelial cells, neurons, and most pertinent for our work, muscle cells and Schwann cells (20–25), and extracellular vesicles are found in most biological fluids including blood, urine, and cerebrospinal fluid (26). However, because there are no procedures for harvesting extracellular vesicles from intact tissue (as opposed to biological fluids), investigators must rely on in vitro approaches to both produce and harvest exosomes from specific cellular populations. We have taken advantage of 2 very well characterized cell lines, the C2C12 muscle cell line and the motor neuron cell line NSC-34 to ask the question: do extracellular vesicles from muscle influence cell survival and/or neurite outgrowth from motor neurons?

## Materials and methods

### Cell culture

All cells were grown in a humidified atmosphere of 5% CO_2_ and 95% air at 37°C.

#### C2C12

The C2C12 skeletal muscle cell line was obtained from the American Type Culture Collection (#CRL-1772). Growth media consisted of Dulbecco's modified Eagle's medium (DMEM) (Cellgro, Manassas, VA) supplemented with 10% foetal bovine serum (FBS; Atlanta Biologicals, Lawrenceville, GA), 4 mM I-glutamine (Gibco, Life Technologies, Grand Island, NY), 1 mM sodium pyruvate (Gibco), 2.5 µg/ml amphotericin B (Fisher Scientific, Fair Lawn, NJ) and 100 U/ml penicillin/100 µg/ml streptomycin (Gibco). Cells were sub-cultured every 3–4 days prior to reaching confluence. To differentiate the cells into myotubes, cultures were placed into differentiation media; AIM-V serum-free medium (Gibco). Myosin expression was used as a marker for cell differentiation as indicated by immunofluorescence and Western blot using standard methods. Primary antibodies were against myosin (Millipore, clone A4.1025) and beta-actin (Santa Cruz C4 sc-47778); secondary antibody was an HRP-conjugate (Jackson, 115-035-003). Conditioned media was harvested between 48 and 96 hours after differentiation, spun at 3,000 g for 10 minutes, and stored frozen at −80°C until use.

#### Extracellular vesicle purification and labelling

Extracellular vesicles were isolated with slight modifications from previously published standard differential centrifugation methods (27). Media from the C2C12 myotube cultures (days 3–4 of differentiation) was thawed and concentrated in a Centricon Plus-70 filter (100 K NMWL, Millipore), spun at 12,000 g for 20 minutes, the supernatant removed and spun at 120,000 g for 70 minutes (Beckman Ti SW-41 rotor). The resulting pellet was re-suspended in Dulbecco's PBS with 20 mM Hepes. Protein concentration was determined using a micro-BCA kit (Pierce Biotechnology, Rockford, IL). Small samples of extracellular vesicles were labelled with PKH26 (Sigma Aldrich, St. Louis, MO) according to the manufacturers specifications in order to visualize extracellular vesicle uptake by the NSC-34 cultured cells. Briefly, purified extracellular vesicles were diluted in 1 ml of diluent C, and then added to 1 ml of the prepared dye (4×10^−6^ M) and incubated at room temperature (RT) for 3 and ½ minutes. To stop the reaction, an excess of protein was added in the form of 2 ml of 1% cytochrome C (Sigma Aldrich) and incubated for 1 more minute. This was then diluted by adding 11 ml of PBS and the labelled vesicles were concentrated in an Amicon 15 spin column (100 K NMWL, Millipore) spun at 3,000 g for 30 minutes at 4°C. The small size of cytochrome C (12.4 Kd) allows it to freely pass through the 100 K NMWL filter. The washing procedure with PBS was repeated 3 times. After the final wash, the exosomes were collected from the spin column and stored at 4°C until used. As a control, a dye-only sample was treated identically.

The presence of CD63 and calnexin was determined by Western blot analysis. CD63 is a common marker for extracellular vesicles and calnexin is a sensitive indicator of the purity of the raw conditioned media since it is a marker for endoplasmic reticulum, which could be present in the conditioned media due to C2C12 cell lysis and might co-precipitate with the extracellular vesicles. Equal amounts of protein from C2C12 cell lysates or extracellular vesicles purified from C2C12 conditioned media were loaded onto Nu Page 4–12% Bis-Tris gels (Life Technologies, California) and processed according to standard procedures; the primary and secondary antibodies for CD63 were from System Biosciences, CA (kit# EXOAB-CD63A-1), the primary calnexin antibody was from Abcam, MA (ab22595) with a secondary antibody HRP-conjugate from Jackson Laboratories (111-035-003), and the primary actin antibody was from Santa Cruz (sc-47778) with a secondary antibody HRP-conjugate from Jackson Laboratories (111-035-003). The extracellular vesicle size range was estimated by the Nanosight instrument (Nanosight Limited, England). Figure 2 illustrates typical nanosight data from 247 tracks that were analyzed as follows: automatic blur, track length and size; detection threshold 8 multi; temperature 24°C; viscosity 0.91 cP; frames per second 13.54; EMCCD camera gain set at 464; measurement time of 90 seconds with one 10-second video recorded; drift velocity 548 nm/s; and a camera shutter of 47 ms.

#### NSC-34

The NSC-34 motor neuron cell line (CELLutions Biosystems Inc., Ontario) was maintained in growth media consisting of DMEM supplemented with 10% FBS, 4 mM I-glutamine (Gibco), 2.5 µg/ml amphotericin B (Fisher Scientific) and 100 U/ml penicillin/100 µg/ml streptomycin (Gibco). Cells were sub-cultured every 3–4 days prior to reaching confluence. To differentiate, the cells cultures were switched to differentiation media consisting of Neurobasal (Gibco), 4 mM I-glutamine (Gibco), 2.5 µg/ml amphotericin B (Fisher Scientific), 100 U/ml penicillin/100 µg/ml streptomycin (Gibco).

#### NSC-34 neurite growth assay

NSC-34 cells were seeded in duplicate onto collagen I coated 8 chamber slides (BD Biocoat, Bedford, MA) at a density of 5.175×10^3^ cell/well in 300 µl in differentiation medium alone (controls) or differentiation medium containing C2C12 myotube extracellular vesicles (200, 100, or 50 ng/µl). These concentrations of vesicles are comparable to a ratio of donor cells to recipient cells of 200:1, 100:1, or 50:1. The concentration of extracellular vesicles (as judged by total protein) was chosen so that on a per-cell basis the extracellular vesicles concentration would be equivalent between the axon outgrowth assay and the viability assay (see below). Slides were incubated for 4 days after which they were fixed with 2% paraformaldehyde (w/v) for 30 minutes at RT and washed 3×5 minutes in PBS. Care was taken during fixing and washing process so as to minimize the number of cells that were dislodged from the substrate. Immunocytochemistry was carried out as follows; nonspecific binding sites were blocked with 10% normal goat serum (Vector Laboratories, Burlingame, CA), 6% bovine serum albumin (Sigma Aldrich), 0.1% Triton X-100 (Promega) for 1 hour at RT. Cells were then incubated in primary antibody 1:500 Neuronal Class III β-tubulin (Clone TUJ1 1-15-79, Covance, Dedham, MA) overnight at 4°C, washed for 3×5 minutes in PBS and incubated in secondary antibody 1:1000 Alexa Fluor 555 goat anti-rabbit IgG (H +L) (Invitrogen, Eugene, OR) for 1 hour at RT. Slides were mounted with Prolong, with DAPI (Invitrogen, Eugene, OR).

Semi-automated analysis of neurite length and complexity was carried out by an unbiased observer using the NeuriteQuant program (28), which is freely available and functions as a plug-in for the ImageJ analysis program. Images were obtained using a 10× lens in a fluorescently equipped microscope, and consisted of every-other visual field of the 8-well chamber. Two separate images were taken at each location, 1 for β-tubulin to visualize neurite growth and 1 for DAPI to visualize the cell nucleus. The images were processed in Photoshop CS6 for analysis using layer stacking of the DAPI and β-tubulin images followed by adjusting the overall levels to minimize background noise to signal. The NeuriteQuant parameters were then optimized to properly select neurite and cell body morphology followed by batch quantification using identical neurite analysis parameters for all cells in the study. NeuriteQuant analysis was carried out for >6,000 motor neurons representing 8 independent repetitions of each extracellular vesicle concentration, using 2 independently prepared batches of extracellular vesicles from conditioned media from 2 independent C2C12 myotube cultures.

#### NSC-34 cell viability assay

NSC34 cells were seeded in quadruplicates at a density of 1.2×10^3^ cells/well in 100 µl in opaque tissue culture treated 96-well plates (CELLSTAR, Greiner Bio-One, Monroe, NC) in either differentiation medium alone, or differentiation medium containing various amounts of extracellular vesicles (46, 23, or 11.5 ng/µl) isolated from media of the 3–4 day differentiated C2C12 myotube cultures as described above. Plates were incubated at 37°C for 4 days and CellTiter-Glo Luminescent Cell Viability Assay (Promega) was performed according to the manufacturer's protocol.

#### Statistics

Multiple group comparisons were first carried out with a General Linear Model (ANOVA, with F- and p-values as indicated) and post-hoc comparisons between groups were carried out with *t*-tests (Fisher least significant difference, p-values as indicated).

## Results

### Time course of C2C12 myosin expression

**Fig. 1:**
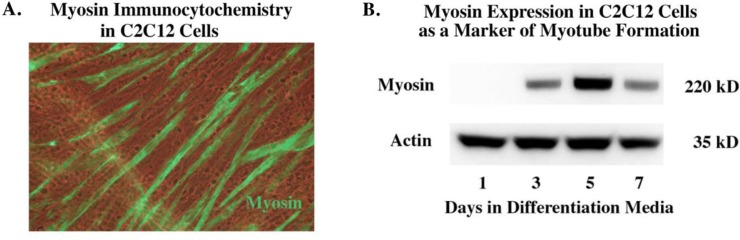
Time course of myosin expression in serum-free AIM-V media (Invitrogen). After propagating cells in DMEM with foetal bovine serum the cells are switched to Aim-V. Panel (A) shows staining for myosin in the early differentiation phase, showing the beginning of myotube formation. Panel (B) shows the expression of myosin begins by day 3 and is still present by day 7.

The differentiation of the myoblast C2C12 cell line into differentiated contractile myotubes (as indicated by myosin expression) is shown in Fig. 1. Myosin begins to be detected by immunohistochemistry and Western blot by day 3 of differentiation. We were specifically interested in extracellular vesicles from differentiated C2C12 myotubes as opposed to non-differentiated myoblasts because we wanted to relate our results to adult motor neuron regeneration rather than developmental aspects that might shape such projections prior to the completion of muscle development.

### Characterization of C2C12 myotube extracellular vesicles

**Fig. 2:**
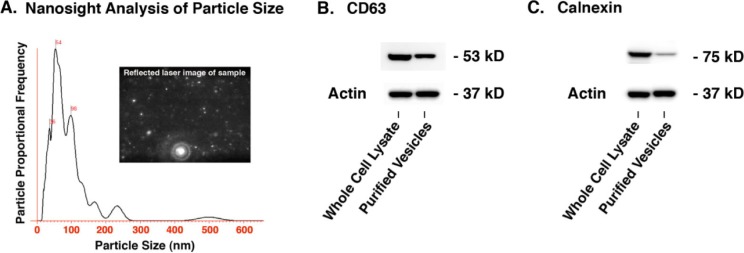
Characterization of C2C12 extracellular vesicles. (A) The Nanosight instrument and nanoparticle tracking analysis revealed vesicles primarily 100 nm and less with a peak around 54 nm. (B) Western blot showing the presence of the tetraspanin CD-63 which is a standard marker for extracellular vesicles (24 µg of total protein loaded per lane). (C) Western blot showing significant reduction of the endoplasmic reticulum marker calnexin in the extracellular vesicle fraction compared to the cell lysate which validates the enrichment procedure that was used to isolate the extracellular vesicles (12 µg of total protein loaded per lane). See Supplementary file for uncropped images of the Western blots.

The range of extracellular vesicles sizes as estimated by the Nanosight instrument was primarily 100 nm and less with a peak around 54 nm. CD63 is often used as a characteristic protein marker for extracellular vesicles whereas calnexin is a marker for endoplasmic reticulum that would be expected to be present in C2C12 cells but significantly reduced in the extracellular vesicle fraction. Figure 2 illustrates that CD63 was present in the extracellular vesicle fraction while calnexin was significantly reduced compared to the cell lysate, thus demonstrating the ability of the purification protocol to effectively enrich extracellular vesicles from other cellular constituents.

### Extracellular vesicle uptake in vitro and NeuriteQuant analysis of motor neuron process outgrowth

**Fig. 3:**
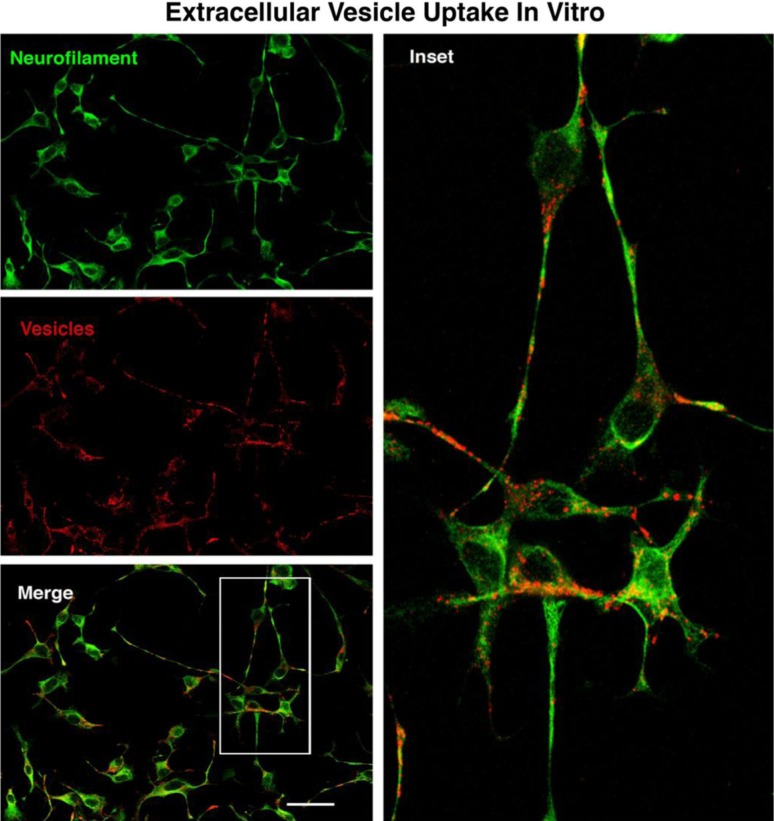
The motor neuron cell line NSC-34 was exposed to fluorescently labelled vesicles for 3 hours, then fixed and stained for neurofilament to label neurite processes. There was extensive uptake of the labelled vesicles by the neurite processes with some indication of accumulation around perinuclear regions. Size bar=50 microns.

**Fig. 4:**
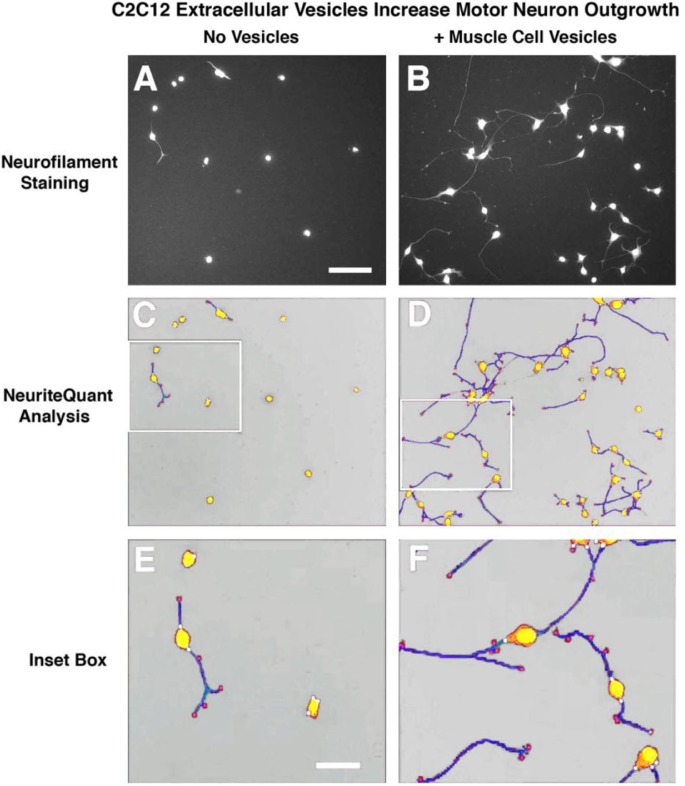
NeuriteQuant analyses of motor neuron process outgrowth. (A and B) Raw images from control (no vesicles) and vesicle-treated cultures. (C and D) Processed image after running NeuriteQuant program. (E and F) Higher power image of inset boxes from C&D to show various aspects of the quantified data. Motor neuron cell bodies are shown in yellow, neurite processes are shown in blue, with their attachment points to the cell bodies indicated by small white circles, and their end points indicated by small red circles. Size bar in A=100 microns and in E=30 microns.

**Fig. 5:**
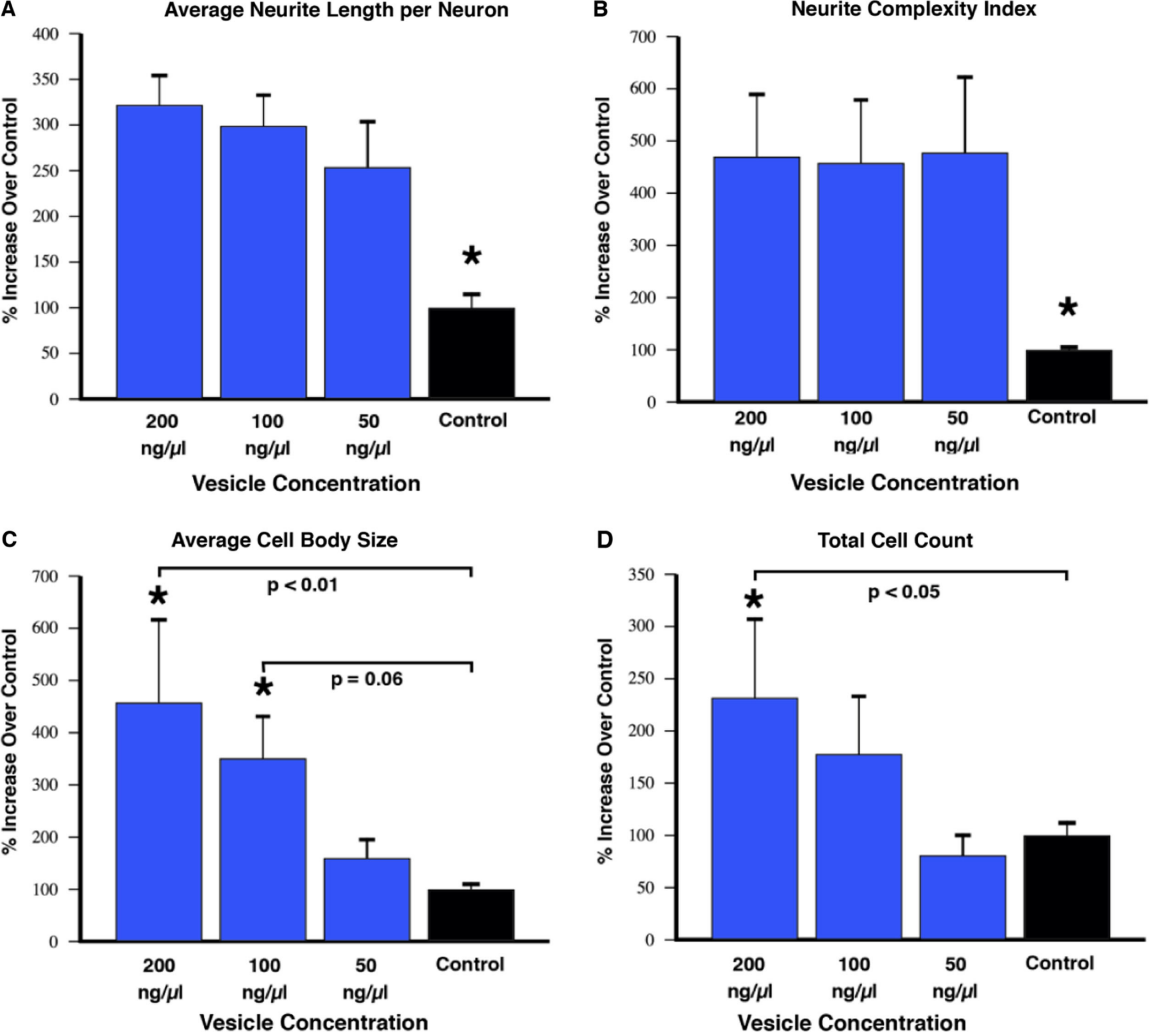
NeuriteQuant analysis (mean±SEM). Motor neuron cells were cultured for 4 days in the presence or absence of varying concentrations of vesicles purified from conditioned media of muscle cells as described in the text. Semi-automated analysis of average neurite length per neuron (A), and the complexity of the neurites as judged by the number of branches per neurite (B) indicates a significant increase in length and complexity for all vesicle-treated groups compared to control (F=0.04, *t*-tests <0.05). (C) There was a dose-response effect on cell body size due to vesicle treatment compared to control that showed a trend at 100 ng/µl (p=0.06) and reached statistical significance with the 200 ng/µl vesicle group (F=0.02, *t*-tests =0.06 and < 0.01, respectively). (D) The total cell count also displayed a dose-response effect that reached significance with the 200 ng/µl vesicle group (F=0.05, *t*-test=0.03). The graphed results for each outcome measure are from conditioned media from 2 independent C2C12 myotube cultures (i.e. 2 biological replicates), with 2 independently prepared batches of extracellular vesicles from each media preparation (i.e. biological replicates), with 8 repetitions of each concentration (i.e. technical replicates). In total, the data represent NeuriteQuant analysis for >6,000 motor neurons. The “N” used for statistics was 4, obtained by the number of biological replicates of vesicle preparations.

Fluorescently labelled extracellular vesicles were used to demonstrate uptake by the motor neuron cells in culture. Motor neurons displayed extensive uptake of the PKH dye-labelled vesicles (Fig. 3) while cultures that received an equal volume of the dye-only retentate from the labelling reaction did not display any PKH fluorescence. Motor neuron process outgrowth was quantified using the NeuriteQuant analysis program. A typical raw image and the corresponding processed image are shown in Fig. 4 for both an extracellular vesicle and control treated sample. The NeuriteQuant program automatically collects data concerning number and size of cell bodies, the number and length of neurite processes, and the complexity of the processes in terms of the number of branch points per process. There was a dramatic effect of extracellular vesicles from the muscle cells on the average neurite length per neuron, the neurite complexity index (number of branches per neurite), average neuronal cell size, and the total number of cells surviving after 4 days in culture (see Fig. 5). Some of these outcome measures demonstrated a clear dose-response effect (e.g. 5C and 5D) and other outcome measures showed significant effects for all doses tested (5A and 5B). In these initial experiments our primary purpose was to “bookend” the effects on outgrowth of NSC-34 cells rather than carrying out exhaustive dose-response curves, although we suspect it is certainly quite likely that additional fractionated doses would demonstrate dose-response effects on all the outcome measures. Pilot experiments demonstrated that changing media in the culture dishes resulted in noticeable loss of the fine neuritic processes, and that culture conditions began to deteriorate at longer survival times. Therefore, we chose a 4-day time point to analyze neurite outgrowth because at that time neurites were clearly present as judged by phase contrast microscopy.

### NSC-34 cell viability assay

**Fig. 6:**
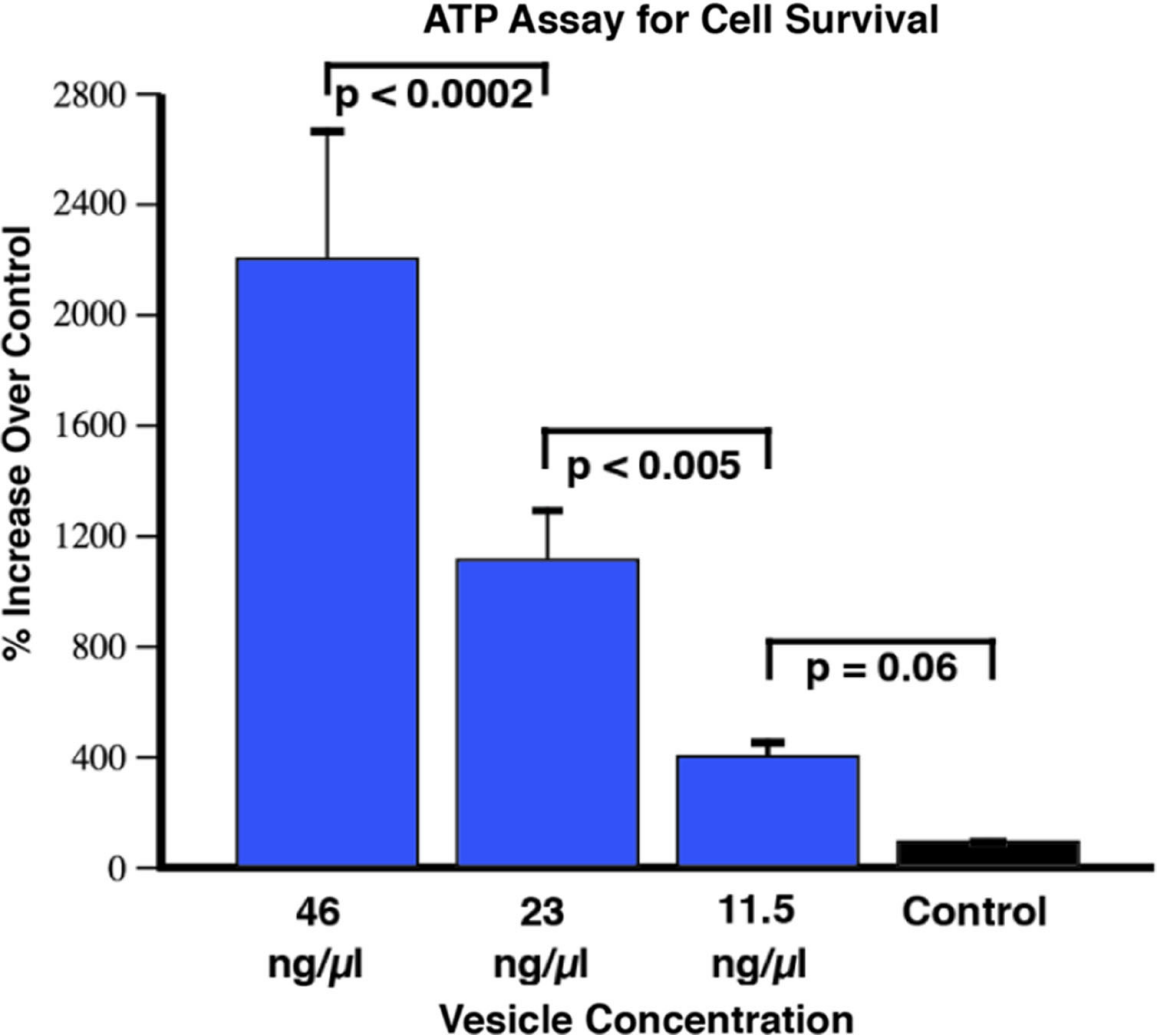
CellTiter-Glo viability assay of motor neurons after 4 days in culture. The control treatment refers to cells grown in media alone, without the addition of vesicles. There was a strong dose-response effect on cell survival compared to control with a trend at the lowest concentration (11.5 ng/µl), and then significantly greater effects with each increased concentration of vesicles (F<0.0001; *t*-tests as indicated on the figure; mean±SEM). The graphed results are from conditioned media from 3 independent C2C12 myotube cultures (i.e. 3 biological replicates), with 2 independently prepared batches of extracellular vesicles from each media preparation (i.e. biological replicates), with 12 repetitions of each concentration (i.e. technical replicates). The “N” used for statistics was 6, obtained by the number of biological replicates of vesicle preparations.

In order to more systematically investigate the cell survival effect of extracellular vesicle addition, we carried out the CellTiter-Glo Luminescent Cell Viability Assay (Promega, Madison, WI) in a 96-well format. This assay uses a luciferase reaction to measure the amount of ATP from viable cells. The amount of ATP in cells correlates with cell viability, because within minutes after membrane disruption cells lose the ability to synthesize new ATP and the endogenous ATPases destroy any remaining ATP. We first examined the robustness of this assay to generate a standard curve that accurately reflected the number of motor neurons plated immediately after determining their number with a hemocytometer. We were able to obtain linear results (r^2^ of 0.99) over 4 orders of magnitude, within the equivalent density range we used for the NeuriteQuant analysis (1.2×10^3^/0.7 cm^2^), and with a reliable detection limit of approximately 15 cells. This assay corroborated the findings from the NeuriteQuant data in terms of there being a dramatic effect on cell survival that was dose dependent (see Fig. 6). The results are from 3 independent 96-well plates for each extracellular vesicle concentration, with each plate being an independent batch of NSC-34 cells, and also representing 2 independently prepared batches of extracellular vesicles from conditioned media from 2 independent C2C12 myotube cultures.

## Discussion

Functional recovery following peripheral nerve repair is largely dependent upon regenerating axons correctly re-innervating their original end organs, e.g. skin and muscle. Our laboratory has a long-standing interest in the field of peripheral nerve repair and the underlying mechanisms that guide the regeneration accuracy of motor neurons back to muscle (29–34). We have championed the hypothesis that muscle, as an end organ, elaborates signals that regenerating motor neurons respond to at a more proximal repair site (see especially (32)). The current in vitro studies were undertaken to examine the possibility that extracellular vesicles from a muscle cell line could influence the outgrowth and survival of a motor neuron cell line.

The C2C12 skeletal muscle cell line has been extensively described previously (35–38). This myoblast-like cell line can be rapidly differentiated into contractile myotubes, which produce characteristic muscle proteins, e.g. myosin. Recent reports have also suggested that this cell line, like most cell lines, secretes extracellular vesicles into the culture media (24). The NSC-34 motor neuron cell line was developed as a fusion between N18TG2 neuroblastoma cells and neuron-enriched embryonic day 12–14 spinal cord cells. This cell line expresses many of the morphological and physiological properties of motor neurons including, extension of processes, formation of contacts with cultured myotubes, synthesis and storage of acetylcholine, support of action potentials, induction of myotube twitching, and expression of neurofilament proteins (39).

The results of the current experiments show that when extracellular vesicles were harvested from differentiated C2C12 cells and applied to the NSC-34 motor neuron cell line there were significant increases in both cell survival as well as neurite outgrowth. Several of these effects were clearly dose dependent, such as effects on motor neuron cell body size and survival (Figs. 5C, 5D, and 6). In terms of the cell survival effects, the 96-well CellTiter-glo assay showed a significant enhancement of cell survival at all 3 of the extracellular vesicle concentrations whereas the NeuriteQuant analysis only showed an effect for the 2 highest concentrations. We consider the Cell Titer assay to be a more sensitive measure of survival because once the cells are plated into the wells nothing is added or removed before adding the assay reagents and reading the plate. For the NeuriteQuant analysis there is always the possibility of losing some cells during the several fixing, washing, and staining steps necessary to carry out the analyses, and thus it may be more difficult to discern small differences. Interestingly, the stimulatory effects on average neurite length and complexity were observed at all of the extracellular vesicle doses used in these studies (Fig. 5A and 5B). Further work will be necessary to determine if this might suggest different underlying mechanisms for these various outcome measures, or if the ability of motor neurons in these conditions to grow a longer and more complex neurite is simply more sensitive to the application of muscle cell extracellular vesicles.

It has recently been suggested that extracellular vesicles secreted by stem cells, rather than the stem cells themselves are the mediators of some of the well-known ability of stem cells to reduce tissue injury and enhance tissue repair (40, 41). This is of interest in terms of recent work showing an enhancement of sciatic nerve regeneration in the rat due to transplantation of mesenchymal stem cells into the nerve lesion (42), and suggests the possibility that just as has been found in other research fields, extracellular vesicles themselves may play a role in nerve regeneration. An additional aspect of extracellular vesicles that makes them ideal long distance messengers is that the internal molecular contents of extracellular vesicles are protected from degradation by trypsin and RNAse as long they remain intact (43, 44). All of these findings suggest that using extracellular vesicles from stem cells instead of the cells themselves could be beneficial because of reduced concerns for safety and inherent limitations due to transplanting viable replicating cells.

The possible role of extracellular vesicles in the field of nerve regeneration is just beginning to be investigated. Our current work in vitro shows that extracellular vesicles from muscle (the target end organ of motor neurons) have significant effects on motor neuron survival and neurite outgrowth. There will certainly be significant challenges in terms of translating such findings into in vivo applications to increase motor neuron regeneration accuracy. However, we are encouraged by recent elegant work from the laboratory of Felipe Court (25), which has shown that extracellular vesicles harvested from cultured Schwann cells enhance in vivo axonal regeneration in the rat sciatic nerve, and the recovery of function as judged by the pinch test. The results of that work clearly demonstrated the possibility of in vitro harvested extracellular vesicles to affect nerve regeneration in vivo. There is also a precedent in the sense that extracellular vesicles from various cell types are already in clinical trials in the tumour biology field (45), and because extracellular vesicles are immunologically tolerated, display targeting ligands on their surface, can cross biological barriers and can be delivered in vivo to selected cell populations (27, 46–48), it is certainly worthwhile to pursue such work in relation to nerve regeneration.
